# Percussion and PSO-SVM-Based Damage Detection for Refractory Materials

**DOI:** 10.3390/mi14010135

**Published:** 2023-01-04

**Authors:** Dan Yang, Yi Peng, Ti Zhou, Tao Wang, Guangtao Lu

**Affiliations:** 1 Key Laboratory for Metallurgical Equipment and Control of Ministry of Education, Wuhan University of Science and Technology, Wuhan 430081, China; 2 Hubei Key Laboratory of Mechanical Transmission and Manufacturing Engineering, Wuhan University of Science and Technology, Wuhan 430081, China; 3 Precision Manufacturing Institute, Wuhan University of Science and Technology, Wuhan 430081, China; 4 Wuhan Digital Engineering Institute, Wuhan 430074, China

**Keywords:** refractory materials, percussion, damage detection, mel spectrogram, support vector machine (SVM), particle swarm optimization (PSO), histogram of oriented gradient (HOG), local binary patterns (LBP)

## Abstract

Refractory materials are basic materials widely used in industrial furnaces and thermal equipment. Their microstructure is similar to that of many heterogeneous high-performance materials used in micro/nanodevices. The presence of damage can reduce the mechanical properties and service life of refractory materials and even cause serious safety accidents. In this paper, a novel percussion and particle swarm optimization-support vector machine (PSO-SVM)-based method is proposed to detect damage in refractory materials. An impact is applied to the material and the generated sound is recorded. The percussion-induced sound signals are fed into a mel filter bank to generate time–frequency representations in the form of mel spectrograms. Then, two image descriptors—the local binary pattern (LBP) and histogram of oriented gradient (HOG)—are used to extract the texture information of the mel spectrogram. Finally, combining both HOG and LBP features, the fused features are input to the PSO-SVM algorithm to realize damage detection in refractory materials. The results demonstrated that the proposed method could identify five different degrees of damage of refractory materials, with an accuracy rate greater than 97%. Therefore, the percussion and PSO-SVM-based method proposed in this paper has high potential for field applications in damage detection in refractory material, and also has the potential to be extended to research on damage detection methods for other materials used in micro/nanodevices.

## 1. Introduction

Refractory materials are composite materials resistant to thermal shock and chemical erosion [1], and their heterogeneity and microstructural complexity have commonalities with related materials [2,3,4] used in micro/nano-devices. Multiple forms of damage in refractory material such as cracks and holes can occur in complex production environments. These damages can reduce the performance of the material and seriously jeopardize the normal operation of devices. Therefore, damage detection for refractory materials after manufacture is vital to ensure their quality and, thus, the stable operation of industrial production.

Currently, several common non-destructive testing (NDT) methods have been attempted to detect damage of certain structures, including the acoustic emission (AE) method [5,6], the ultrasonic method [7,8], the radiography method [9], etc. AE is the phenomenon of transient elastic waves generated by the rapid release of local energy within a material (or structure). Liu et al. [10]. proposed a damage detection method of refractory materials using principle component analysis and the Gaussian mixture model to reduce the dimensions of the relevant parameters of the AE signal and describe the overall properties of material damage. The complex structure of refractory materials produces extremely complex AE signals, which makes damage classification difficult. The ultrasonic method detects the damage state by accurately analyzing changes in the ultrasonic signal characteristics [11,12]. Due to the anisotropy of refractory materials, ultrasonic testing methods have a low signal-to-noise ratio in applications, and it is difficult to obtain useful damage information from the signals [13]. The radiographic method adopts a negative as a recording medium, which can directly obtain a direct visual image of the damage [14]. This method has a high detection rate for volumetric defects (porosity, inclusions). However, it has difficulty detecting cracks in the direction perpendicular to the rays and is harmful to human health [15]. Each of these methods has its drawbacks. Therefore, it is necessary to seek a simple, safe, economical and implementable damage detection method for refractory materials.

The percussion method, as a convenient NDT technique, can distinguish structural damage through percussion-induced sounds [16,17,18,19]. In traditional tapping testing, inspectors cannot recognize the nuances of sounds due to the limited frequency range of hearing. Recently, improvements in computer performance and the application of artificial intelligence technology in many fields has provided the possibility to overcome this limitation [20,21,22]. Kong et al. [23] proposed a novel percussion-based method to detect bolt looseness. The method utilized a microphone to record sound. Power spectrum density (PSD) values were adopted as features of the sound data and the method successfully achieved the evaluation of bolt looseness using a decision tree model. Cheng et al. [24] proposed a detection method using percussion and voice recognition. A microphone was used to record sound signals from tapping different sand depositions in pipelines and MFCC features of the tapping data were extracted to achieve the identification of deposition volume in pipelines. Furthermore, researchers have detected looseness of cup-lock scaffolds [25], internal cavities of timber columns [26] and moisture of concrete [27] by processing and analyzing percussion-induced sound signals. These studies indicate that the percussion-based method is user-friendly and has reasonable detection accuracy. Nevertheless, investigation of the quantitative relationship between damage to refractory materials and percussion sound signals is lacking.

The latest research [28,29] tends to convert sound signals into time–frequency representations (TFRs), such as short-time fourier transform (STFT) spectrograms and constant-Q transform (CQT) spectrograms, which depict the energy of the signal in different frequency bands over time. STFT spectrograms [30] are low in computation and offer frequency components in linearly spaced frequency bands; however, they are not conducive to highlighting spectral information in the low frequency range. Mel spectrograms compress the frequency range by nonlinear mapping after STFT processing, which can improve the resolution of the frequency components in the low frequency range. On the other hand, many recent studies have treated TFRs as texture images and used computer vision techniques on TFRs for sound signal classification [31,32]. The texture features extracted from mel spectrograms can capture the discriminant patterns of sound structure in time and frequency. Local binary patterns (LBPs) [33] and histogram of oriented gradients (HOG) [34] are two representative texture descriptors. HOG deals with the distribution of gradients in different directions and is suitable for dealing with random textures, while LBP deals with pixel intensities and texture microstructures. The feature extraction behavior of different texture descriptors is complementary and the fusion technique [35] yields a significant difference in TFR, achieving recognition results with higher accuracy. Mel spectrograms such as TFRs, LBP and HOG are fused together to extract valid texture information, which is beneficial for analyzing percussion-induced sound signals.

In this study, an easy-to-implement and efficient novel percussion method is used to detect damage to refractory materials. During detection, the percussion-induced sound signals are first transformed into mel spectrograms, which can depict the singularity of different signals. LBP and HOG methods are used to extract the unique textural features of the mel spectrogram to further uncover the hidden damage-related information. Thereafter, the feature vector of the signal is obtained by the fusion of HOG and LBP features. Finally, the PSO-SVM classifier is used to identify the degree of damage to the refractory materials. The rest of this paper is organized as follows. Section 2 provides the methodologies and related principles of the proposed method. Section 3 describes the experimental device and experimental procedure. Section 4 discusses the identification results of the method and provides a comparative analysis with other strategies. Section 5 is the conclusion of the paper.

## 2. Methodologies

**Figure 1:**
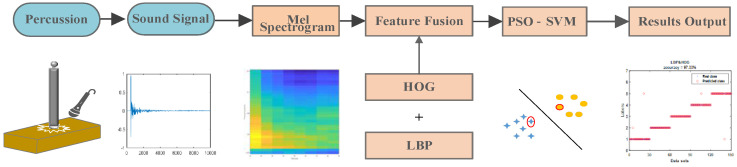
Schematic diagram of the proposed percussion method and PSO-SVM.

The schematic diagram of the proposed percussion method is shown in Figure 1. The method involves three main steps: percussion signal acquisition, feature extraction and damage classification and recognition based on PSO-SVM. Firstly, percussion signals are collected for five different degrees of damage to refractory materials. Then, in order to reveal the variation pattern of the percussion sound signals, the mel spectrogram was used to reflect a large amount of feature information of the signals. In addition, the HOG feature and LBP feature were adopted to capture the texture information of the mel spectrogram variations. Finally, the combination of LBP and HOG was entered into the PSO-SVM classifier, and the output of the PSO-SVM represented the degree of damage to the refractory material.

### 2.1. Feature Extraction

#### 2.1.1. Mel Spectrogram

The mel spectrogram [36] is a combination of the mel scale and a spectrogram. The acquisition process is simple; the input signal is preprocessed (framing, windowing), fast Fourier transform (FFT) is applied and the signal is passed through a mel filter bank.

#### 2.1.2. Histogram of Oriented Gradient

HOG is an excellent local feature descriptor proposed by Dalal and Triggs at CVPR [37,38] for obtaining image feature vectors. The specific steps are as follows:

(1) Grayscale the input images;
(2) Perform color space normalization of grayscale images by the Gamma correction method

(1) $$I(x,y)=I{\left(x,y\right)}^{\gamma }$$

(3) Calculate the gradient (direction and intensity distribution) of each pixel to further obtain the image contour information.
  (2) $$G_{x}(x,y)=I(x,y+1)-I(x,y-1)$$
  where *G_x_*(*x,y*), G_y_(*x,y*) and *I*(*x,y*) denote horizontal gradient, vertical gradient and pixel value, respectively. In addition, the direct and intensity distributions are as follows:
  (3) $$G(x,y)=\sqrt{G_{x}{\left(x,y\right)}^{2}+G_{y}{\left(x,y\right)}^{2}}$$
  (4) $$\alpha (x,y)=\arctan\frac{G_{x}(x,y)}{G_{y}(x,y)}$$

(4) Divide the image into small cells; then count the gradient histograms of each cell and obtain the statistics of gradient distribution in different directions;
(5) Several cells form a block. HOG features of all blocks of the image are obtained through a sliding block.

#### 2.1.3. Local Binary Patterns

LBP is a simple and efficient method for texture description [39] that is able to obtain a binary pattern of differences between a central pixel and neighboring pixels. Specifically, the pixels of the image are labeled by thresholding the 3 × 3 neighborhood of each pixel with the center value and using the result as a binary number. The central pixel value is calculated as follows:

(5) $$LBP(x_{c},y_{c})=\sum_{p=0}^{p-1}2^{p}S(i_{p}-i_{c})i_{p}i_{c}$$

where *i_c_* is the grayscale of the central pixel, the grayscale of the neighbourhood in the pixel matrix is *i_p_* and *S* is the step function, as shown below:

(6) $$S\left(x\right)=\{{}_{0,x<0}^{1,x\geq 0}$$

### 2.2. Classification

#### 2.2.1. Support Vector Machine

Support vector machine (SVM) is one of the best algorithms in machine learning and is commonly used to solve classification and regression problems [40,41,42]. The data points for each sample can be expressed as {*x_i_,y_i_*}, where *i =* 1, 2, 3, …, *N*, *x_i_* ∈ *R^n^*, *y_i_* ∈ {+1,−1}. The expression of the hyperplane in the linearly divisible case can be written as:

(7) $$f(x)=w^{T}x+b=0$$

where *f*(*x*) is the separating hyperplane, the parameter *w* is the weight and *b* is the bias. When encountering a linearly indistinguishable problem, the above equation needs to be extended by adding the slack variable *ξ_i_* and the penalty *c* factor to obtain the model for solving the nonlinear problem, as follows:

(8) $$\min\frac{1}{2}{\left\|w\right\|}^{2}+c\sum_{i=1}^{N}\xi_{i},s.t.y_{i}(w\cdot x_{i}+b)+\xi_{i}-1\geq 0,\;\xi_{i}\geq 0$$

This equation is then transformed into a pairwise problem using the Lagrange multiplier method to obtain the decision function as follows:

(9) $$f(x)=\mathrm{sgn}\left(\sum_{i,j=1}^{N}\alpha_{i}y_{i}k(x_{i},x_{j})+b\right)$$

where *α_i_* is only one variable included in the Lagrangian function; *k*(*x_i_,y_i_*) = φ(*x_i_*)^T^φ(*x_j_*) is the kernel function introduced to address the difficulty of direct calculation due to the high dimensionality of the features.

#### 2.2.2. Particle Swarm Optimization Algorithm

The PSO algorithm [43] is inspired by the predatory behavior of a flock of birds searching for food randomly and is used to solve optimization problems. PSO is initialized as a flock of random particles; then, the optimal solution is found by iteration. At each iteration, the particles update their velocity and position towards the individual extremes and the global extremes. The update formula is as follows:

(10) $$V_{id}^{k+1}=wV_{id}^{k}+c_{1}r_{1}(P_{id}^{k}-X_{id}^{k})+c_{2}r_{2}(P_{id}-X_{id}^{k})k$$

(11) $$X_{id}^{k+1}=X_{id}^{k}+V_{id}^{k}$$

where *w* is the inertia weight, *k* is the number of current iterations, *c*_1_ and *c*_2_ are learning factors and *r*_1_ and *r*_2_ are uniform random numbers in the range of (0, 1).

#### 2.2.3. The PSO-SVM Method

The RBF kernel function is used in SVM to solve the nonlinear classification problem; classification accuracy is mainly influenced by the penalty factor *C* and the kernel parameter *g*. The PSO algorithm is adopted to search for the ideal parameters *C* and *g* of SVM to achieve the accurate and reliable detection of damage in refractory materials. The specific process of PSO-SVM is as follows:

(1) The specific implementation steps of the PSO-SVM algorithm are as follows. Set the relevant parameters: the population size is 20, the inertia factor is 0.6, the acceleration constants are 1.5 and 1.7, and the number of iterations is 50;
(2) Prepare the train and test data sets. The training sets adopt the five-fold cross-validation method and the classification accuracy is set to the particle fitness value;
(3) The penalty parameter *C* and kernel parameter *g* are [–4, 4]; in addition, the velocity and location of each particle are randomly initialized;
(4) Calculate the adaptability value of each particle and calibrate it;
(5) Update particle velocity and position;
(6) Determine if the termination condition is reached; if so, stop the update; otherwise, return to step (5);
(7) When the number of iterations reaches the initial setting value, the optimal parameters *C* and *g* are obtained.

**Figure 2:**
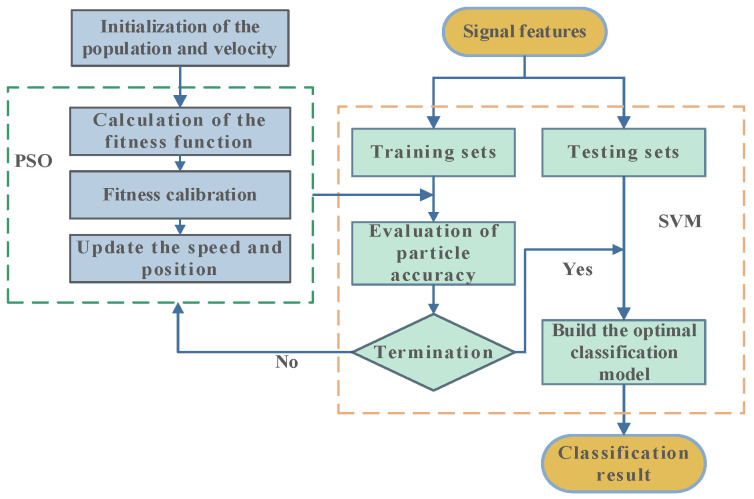
Flow chart of PSO-SVM.

The PSO-SVM model construction process is shown in Figure 2.

## 3. Experimental Setup and Procedure

**Figure 3:**
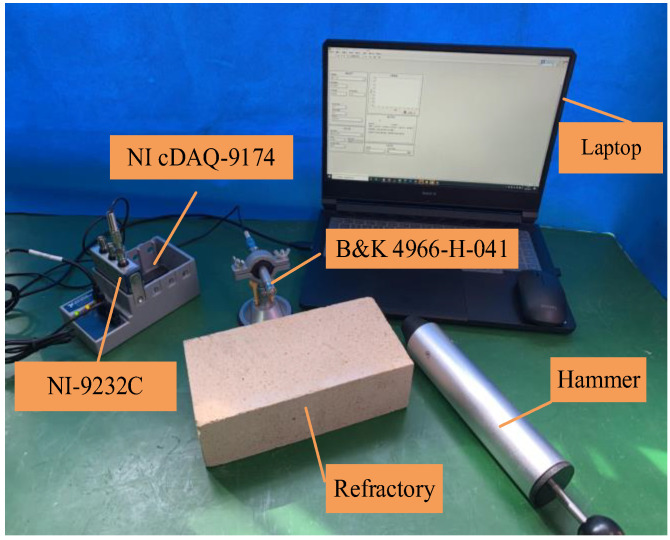
Schematic of the experimental setup.

The samples and devices used for the experiments are given in Figure 3. The high alumina refractory material was manually impacted using an impact hammer, and the percussion-induced sound was captured by a microphone (B&K Type 4966-H-041). The sound signals were collected by a data acquisition system (a NI cDAQ-9174 chassis with a NI-9232C data acquisition module) and stored on a laptop. The microphone was placed at a fixed position approximately 5 cm from the material and the sound signal was acquired at a sampling rate of 100 kHz.

**Figure 4:**
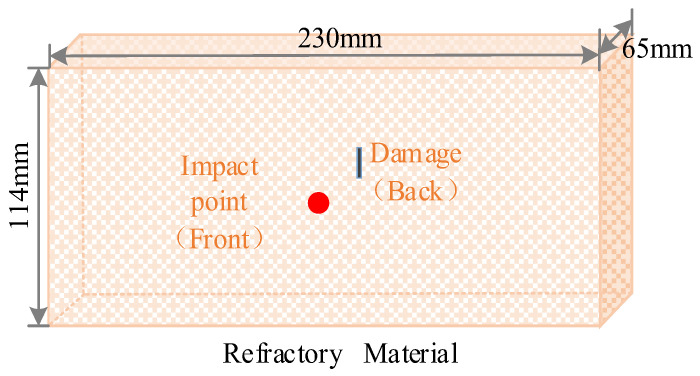
Location of the impact point and the simulated damage.

The size of the refractory material was 230 × 114 × 65 mm. During the experiment, a saw was used to cut slits of different depth on the refractory material to simulate five different degrees of damage (denoted as D1, D2, D3, D4, D5) to the refractory material. The specimen was percussed 100 times for each degree. The locations of the impact point and the simulated damage are shown in Figure 4. The degree of damage in the refractory materials in the impact test is given in Table 1.

## 4. Experimental Results and Analysis

**Figure 5:**
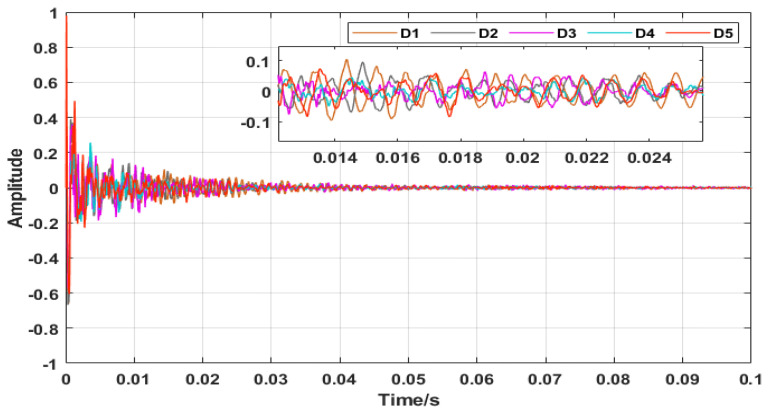
Samples of sound signals taken for five damage degrees.

In the experiment, all percussive sound signals were pre-processed by normalization, and the length of each signal was 0.1 s (sample points = 0.1 × 100,000 = 10,000). Figure 5 depicts the sound signal samples for each of the five damage degrees. It can be seen that the trend of the signals is similar in the time domain.

**Figure 6:**
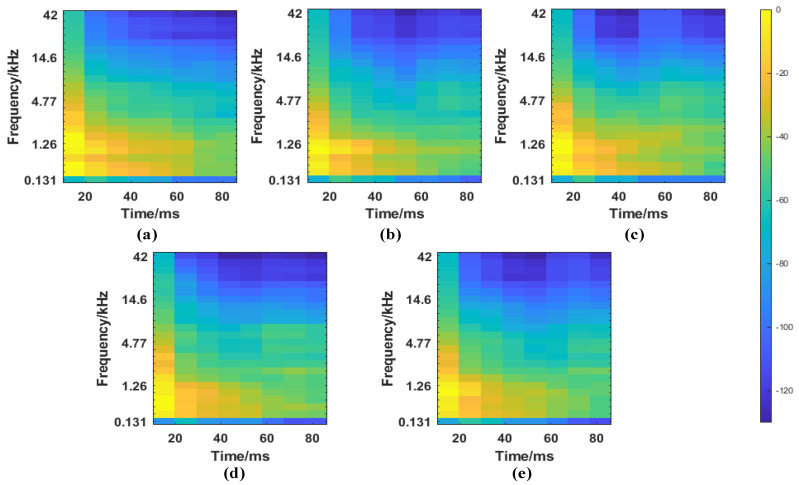
The mel spectrograms of selected signals: (**a**) D1, (**b**) D2, (**c**) D3, (**d**) D4 and (**e**) D5.

Time−frequency analysis was used to convert the signals into mel spectrograms. In mel spectrogram representation, the Hamming window is considered, and the parameters window length and overlap length [44] are set to 2048 sample points and 1024 sample points, respectively. Figure 6 shows the extracted mel spectrogram features. From the figure, it can be seen that the mel spectrogram depicts the energy variations of different frequency bands over time, and that the mel spectrogram representation of sound signals has texture. This texture can be used to differentiate the percussion-induced acoustic signals with different degrees of damage.

**Figure 7:**
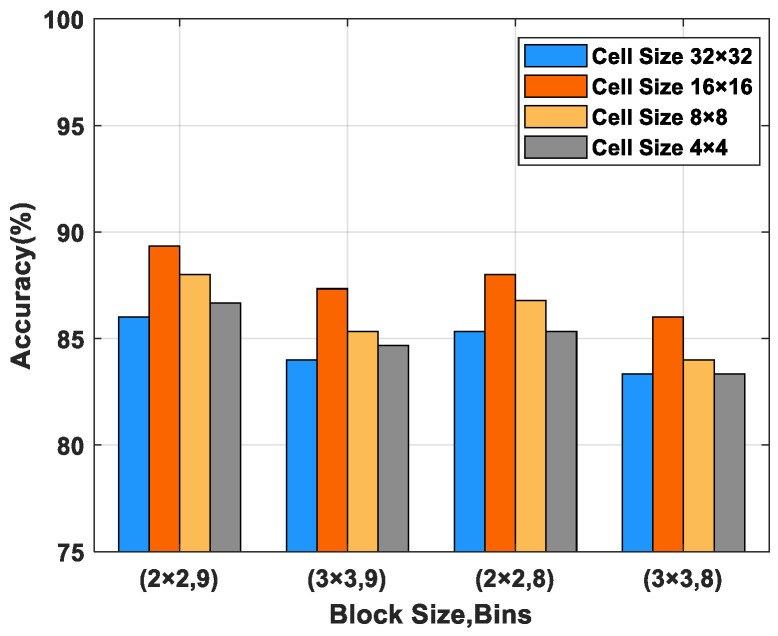
Performance of HOG with different parameters.

**Figure 8:**
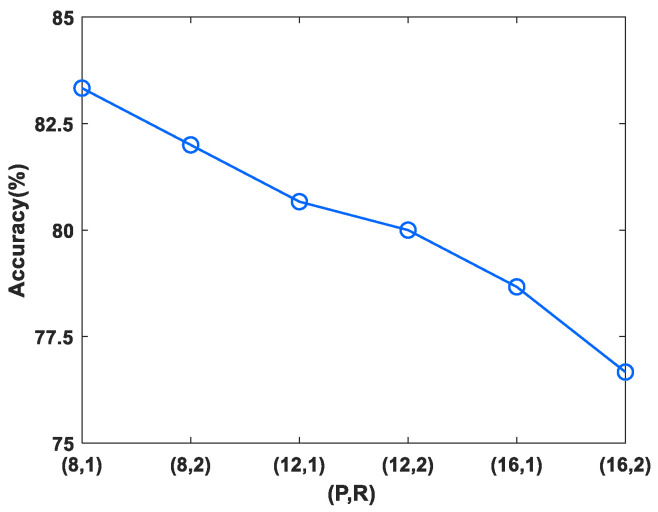
Performance of LBP with different (P, R).

Next, two powerful texture descriptors−LBP and HOG−were considered to extract features from the mel spectrogram. Since the generation of HOG feature depends on cell size, block size and the number of bins [37], these three parameters are discussed in this paper, as shown in Figure 7. In general, the size of the cell has a greater influence on texture information encoding. As illustrated in Figure 7, when the cell size was 16 × 16, the highest recognition accuracy was obtained, followed by 8 × 8. The cell size of 32 × 32 generated the worst performance. In addition, in contrast to the other three options, setting the block size and bins to 2 × 2 and 9 yielded better performance. Therefore, in this work, the parameters of the HOG algorithm were set as shown in Table 2. On the other hand, different sampling radii R and numbers of sampling points P result in different image texture extraction capabilities for LBP features [39]. Six (P, R) pair values were chosen; the accuracy of the LBP features with different parameters is shown in Figure 8. As R becomes larger and the number of P increases, the texture description capability of LBP decreases. It is obvious that the best feature extraction is achieved when R = 1, P = 8. The LBP and HOG features emphasize the different texture information of the mel spectrogram. As the features are complementary, fusion was applied to concatenate the LBP and HOG feature vectors into enhanced vectors (LBP&HOG).

After that, the entire dataset was divided into a training set and a test set. The overall speed and accuracy of the model is closely related to the reasonable partitioning of the data set. Table 3 presents the accuracy and time of different training-to-test set ratios. As can be seen from the table, the best accuracy and fastest speed were achieved when the training-to-test set ratio was 7:3. The total data size for the five damage degrees was 500. Therefore, for each damage degree, 70% of the data from the data sets were randomly taken as the training set, and the other 30% of the data were used as the testing set.

The features obtained in the previous step were used as the input of PSO-SVM and three damage detection models (HOG&LBP + PSO-SVM, HOG + PSO-SVM, LBP + PSO-SVM) were trained. Table 4 shows the optimal parameter values obtained by PSO searching in the solution space. The trained models were used to perform recognition on the test samples.

**Figure 9:**
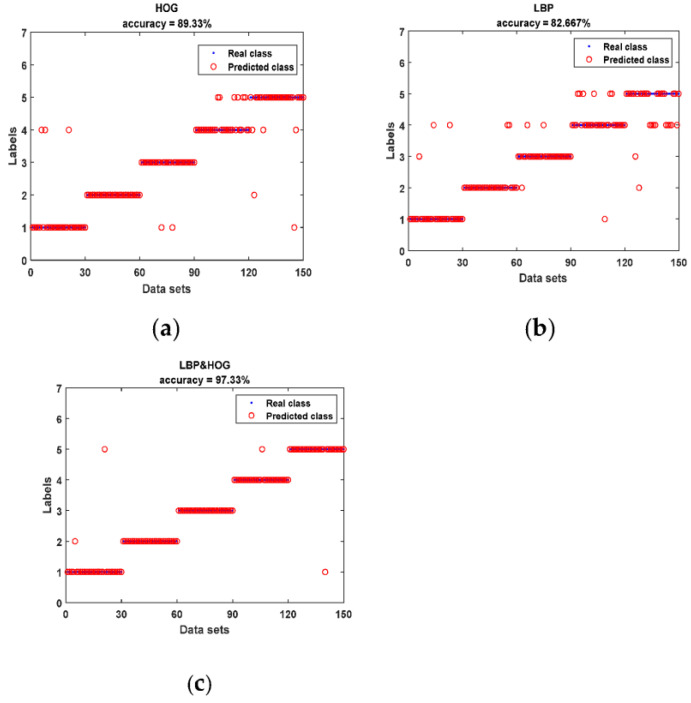
The damage identification results: (**a**) LBP, (**b**) HOG and (**c**) LBP&HOG.

The details and visualization of the predicted and real classes are shown in Figure 9. In Figure 9, the horizontal coordinate indicates the data set, and the vertical coordinate indicates the degree of damage in the refractory material. The ordinates of 1, 2, 3, 4 and 5, respectively denote D1~D5, which are also shown in Table 1. From the figure, it can be seen that the accuracy of HOG features is 89.33%, the accuracy of LBP features is 82.67% and the accuracy of LBP&HOG features is 98.67%. Meanwhile, Figure 9c shows that only four cases in the test set are classified into incorrect classes. It is obvious that the fused features (LBP&HOG) as input outperformed the typical single features in terms of classification performance.

The quality of the output of the PSO-SVM classifier was evaluated by the performance parameters [45] precision, recall, F1-score and error rate in the classification task, as shown in Table 5.

It is observed from Table 5 that the output of the PSO-SVM model had a precision of 0.94–1, a recall of 0.93–1, an F1-score of 0.95–1 and an error rate of 0–0.05. The results demonstrate that the PSO-SVM method yields excellent detection of damage severity in refractory materials.

**Figure 10:**
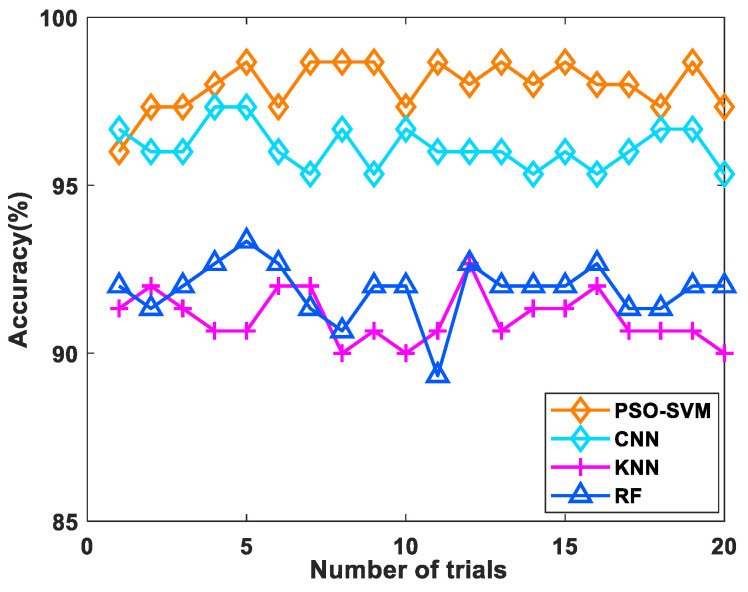
Comparison of classification accuracy with various algorithms.

To assess the effectiveness and superiority of the method proposed in this paper, some recognition results of well-known classifiers were used to the data. These included k-nearest neighbor (KNN), random forest (RF) and convolutional neural network (CNN). The results after repeated experiments are shown in Figure 10. The mean accuracy and implementation time for 10 experiments were calculated, as shown in Table 6. In the KNN classifier, several experiments were performed with various K values. The best performance was obtained with K = 12; however, the recognition was poor at only 91.06%. For the RF classifier, the number of trees was set to 100 and a recognition rate of 91.87% was obtained. Comparing the recognition results of the four classifiers, the proposed PSO-SVM classification is the best; it had the highest average accuracy, achieving a maximum recognition accuracy of 98.67%. The performance of CNN was second, with a maximum accuracy of 96.13% and an average accuracy second only to PSO-SVM. However, with CNN, due to the presence of a large number of convolutional operations, the number of calculation operations for trainable parameters increases significantly, which leads to a longer implementation time the worst performance in terms of implementation speed. As a result, it is proven that the method proposed in this paper obtains the best classification accuracy and saves valuable computational resources with its relatively low time required to complete the recognition task.

**Figure 11:**
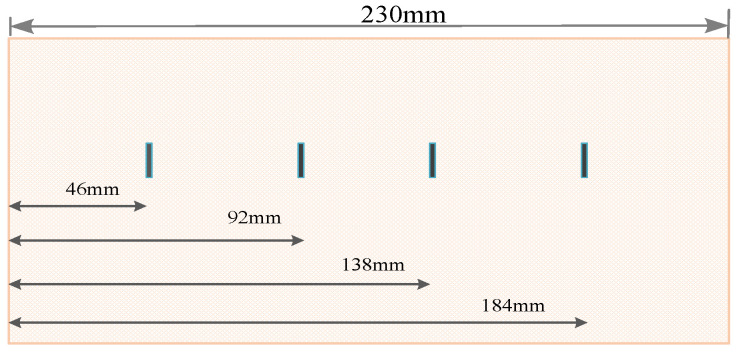
The distribution of damage at different locations.

To further investigate the effect of damage distribution at different locations on the performance of the proposed method, extended experiments were conducted. The shape of the refractory material is symmetrical. With the left side as the baseline, slits with a depth of 5 mm were fabricated at 46 mm, 92 mm, 138 mm and 184 mm (denoted as L1, L2, L3, and L4) from each of the four specimens to simulate damage at different locations. The distribution of damage at different locations is shown in Figure 11.

**Figure 12:**
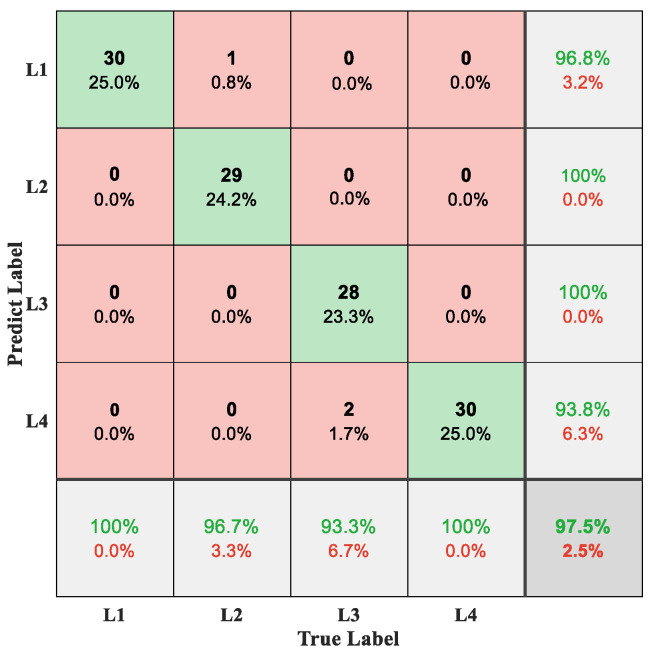
Confusion matrix of different location identification results.

The percussion method was used to obtain percussion sound signals for the damage at different locations. Similarly, mel spectrogram and HOG&LBP were used to process each sound signal to acquire the feature vector. The PSO-SVM algorithm completed the classification detection. All parameters were set in accordance with the best settings obtained from the analysis. A confusion matrix was used to visualize the recognition results of different damage locations, as shown in Figure 12.

In Figure 12, the horizontal and vertical coordinates represent the true and predict labels of different damage locations, respectively. As shown in Figure 12, only a small number of samples were incorrectly identified (e.g., one L2 sample was classified as L1 and two L3 samples were classified as L4), while the rest of the samples were classified to the correct categories. The overall identification accuracy was 97.5%. From the results, it can be seen that the method has good generalization ability and can achieve effective recognition of damage with different location distributions. Therefore, the method proposed in this paper can accurately extract the key features of damage and realize the accurate detection.

In addition, the performance results of the proposed method were compared with other newly published methods for solving the damage detection problem. As can be seen in Table 7, the proposed method in this paper yields better accuracy scores than multiple data processing methods and classification models.

## 5. Conclusions

Based on percussion and PSO-SVM, a novel method for damage detection and identification in refractory materials is proposed in this paper. Sound signals generated by manually controlled percussions are converted to mel spectrograms, and LBP features and HOG features are extracted from mel spectrograms. Then, the two features are fused and input into a PSO-SVM for training to realize damage detection in refractory materials. The experimental results verified the effectiveness of the proposed method. It is worth noting that converting percussion-induced acoustic signals into mel spectrograms and using the fused HOG&LBP method achieved better damage detection in refractory materials than typical single LBP and HOG texture features. Furthermore, the recognition accuracy of the PSO-SVM was in the range of 96–98.67%, with a more stable classification performance and higher classification accuracy than the other three classification strategies. Overall, the damage detection method for refractory materials proposed in this paper is convenient and reliable, with potential for field application. In future work, the combination of robotics and machine learning can be further implemented. Specifically, a robotic arm will carry a tapping device and a microphone for automatic tapping and signal acquisition to produce a system for automatic detection of refractory material damage. In addition, environmental noise is not considered in the paper; further investigation will be conducted on this issue.

## Figures and Tables

**Table 1:** Degree of damage to refractory materials during the percussions.

Damage Degree	D1	D2	D3	D4	D5
Slit depth (mm)	0 mm	5 mm	10 mm	15 mm	20 mm

**Table 2:** Characteristic parameters of HOG algorithm.

Parameters	Value
Cell	16 × 16
Block size	2 × 2
Number of bins	9

**Table 3:** Results of different training and testing ratios.

Ratio (Training Set: Testing Set)	Accuracy (%)	Time (s)
5:5	96.8	26.5
6:4	95.5	30.0
7:3	98.7	23.3
8:2	97.0	41.6
9:1	96.0	52.9

**Table 4:** The values of the optimized parameters.

Method	*C*	*g*
LBP	14	1.0366
HOG	5.6569	0.1768
LBP&HOG	2.8284	0.0625

**Table 5:** The common evaluation metrics results of PSO-SVM.

	Precision	Recall	F1-Score	Error Rate
D1	0.97	0.93	0.95	0.05
D2	0.97	1	0.98	0.02
D3	1	1	1	0
D4	1	0.97	0.98	0.02
D5	0.94	0.97	0.95	0.03

**Table 6:** Classification results under different algorithms.

Algorithm	Time (s)	Max Acc. (%)	Min Acc. (%)	Mean Acc. (%)
PSO-SVM	21.3	98.67	96.00	97.97
CNN	129.6	97.33	95.33	96.13
RF	26.8	93.33	89.33	91.87
KNN	14.5	92.67	90.00	91.06

**Table 7:** The results of the proposed method compared with existing methods.

Authors	Features	Classifiers	Accuracy
Cheng et al. [24]	Mel-frequency cepstral coefficients (MFCCs)	SVM	91.59%
Kong et al. [23]	Power spectrum density (PSD)	Decision tree	92.00%
Yuan et al. [19]	Improved multiscale sample entropy (IMSE)	BP neural network	96.00%
Proposed method	Mel spectrogram—HOG&LBP	PSO-SVM	98.67%

## Data Availability

Due to the nature of the research, the data of this study are not shared publicly and are only available upon reasonable request.
